# The New Anæsthetic

**Published:** 1884-12

**Authors:** A. W. Calhoun

**Affiliations:** Professor of Diseases of Eye, Ear and Throat, Atlanta Medical College, Atlanta, Ga.


THE ATLANTA

### MEDICAL AND SURGICAL JOURNAL.

VOL. I. DECEMBER, 1884. No. 10.

### Original Communications.

### THE NEW ANÆSTHETIC.

A SHORT EXPERIENCE WITH HYDROCHLORATE OF COCAINE AS A LOCAL ANÆSTHETIC.

BY A. W. CALHOUN, M.D., PROFESSOR OF DISEASES OF EYE, EAR AND THROAT, ATLANTA MEDICAL COLLEGE, ATLANTA, GA.

Case 1. J. C. J., S. C., aet. 43, has had stricture of the left nasal duct for some years, but from recent cold an abscess formed in the lacrymal sac, rupturing externally, leaving a fistulous opening just below the internal canthus. When first seen the swelling and pain in the parts were great, so that means had to be used to reduce them with the view to operating.

On Nov. 7th, three drops of a 4 per cent. solution of muriate of cocaine were dropped upon the mucous surface of the lower lid every four minutes until four applications had been made. After an interval of four minutes from the last application, the canalliculus was slit and the knife passed through the entire nasal duct. The patient complained of an uncomfortable feeling, but said it was not a pain. The success in this case is decided, inasmuch as the parts were exceedingly painful to the touch immediately preceding the first application of the cocaine. The pupil enlarged and remained so for several hours.

Case 2. November 7, 1884, Miss S. C., aet. 13, Augusta, Ga., convergent strabismus of the right eye from small central corneal opacity, which resulted from "sore eyes" several years ago. Three drops of the muriate of cocaine (4 per cent.) were instilled at intervals of five minutes until it had been used three times. Waiting five minutes after the last instillation, and finding the cornea could be touched by my finger and the conjunctiva grasped by forceps without the slightest discomfort, the operation was completed without a complaint on the part of the patient, except that she "felt a pressure on top of the head." Pupil dilated and remained so for about eight hours.

Case 3. November 7, 1884, W. H. M., aet 40, Ga. Dislocated cataractous lens in the anterior chamber of the left eye. The ball became sensitive and inflamed. The patient is extremely nervous and debilitated, fainting on two occasions while talking about his case. Three drops of the 4 per cent. solution of the cocaine were dropped upon the inside of the lower lid every four minutes, four times, and after waiting five minutes, the lens was successfully extracted by the lower flap operation. The puncture and counter-puncture were as painless as grasping the conjunctiva, but he suffered some pain as the knife was drawn to and fro in making the flap. It should have been tested more thoroughly in this case on account of the excessive nervousness of the man. Pupil dilated.

Case 4. November 8, 1884, Miss S. E., aet. 16. Double convergent strabismus. The cocaine was used on the right eye as in the other cases and the operation made without pain—"only an uncomfortable feeling." Pupil dilated for five or six hours.

Case 5. November 9, 1884, P. F., aet. 60, North Carolina. Mature senile cataract left eye. The cocaine used in the same manner, except more (five) times. When touching the cornea and pulling upon the ball with the forceps were not felt, then Graefe's linear extraction was easily completed in all its details, and when asked as to the amount of pain he suffered, the patient replied that "it was less than a pin scratch."

Case 6. November 10, '84, S. E. M. aet. 38, North Carolina. Large pterygium growing from the external canthus of the right eye and covering the temporal half of the pupil. Used the cocaine as before. Cutting the base of the pterygium and putting in the sutures were more or less painful, but otherwise without pain.

Case 7. November 11, '84. Same person as case 4, but left eye. Convergent strabismus. The same course was pursued as formerly, and the patient said the operation gave "slight pain," though she gave but little evidence of it while undergoing the operation.

Case 8. November 14, '84, Miss S W., aet. 50, Alabama. Mature senile cataract in both eyes. Used the cocaine six times at intervals of four minutes and made Graefe's linear extraction on the left eye without any pain whatever.

Case 9. November 14, '84, John R., aet 60, Florida. Internal strabismus right eye from paralysis of external rectus muscle. Under the influence of the cocaine the internal rectus was cut and the external rectus muscle was advanced with only a slight inconvenience to the patient. The delicate health of the man and the usual painful tediousness of the operation was an excellent test of the remedy.

Case 10. November 14, '84, A. P., aet. 30, Florida. Pterygium upon the right eye. The cocaine used and operation made, but with about the same pain as in case six. I have tested it very patiently and thoroughly upon a fourteen year old boy, who has a polypus in the larynx, applying it with a brush, at short intervals, for more than an hour, directly to the growth in the wind pipe, but upon attempting to operate, I found the same sensitiveness which had formerly baffled me in attempting to grasp the tumor. While these are only a few cases, they show unmistakably the great power of the medicine, and it is to be hoped that future experience will still more thoroughly demonstrate its efficacy as a local anæsthetic. That it will disappoint us in many instances we cannot doubt, and that it is suitable for only superficial operations is already clearly shown. Before growing too enthusiastic, it would be well to await more extended experiments and experience, though at present it bids fair to become a boon to our fellow-sufferers.

